# Program for disordered eating in a primary care setting

**DOI:** 10.1186/2050-2974-1-S1-O25

**Published:** 2013-11-14

**Authors:** Suzi Scholey

**Affiliations:** 1 Headspace Gold Coast, Australia

## 

The Program for Disordered Eating run at Headspace Gold Coast is a new intervention developed in September 2012 in response to an identified unmet need for a service for young people with eating issues. This program aims to provide screening, assessment and early intervention for those who are suffering from disordered eating at a distressing level but who do not meet the criteria for local public health eating disorder services. Headspace is an ideal organisation as it targets young people between 12 and 25 and the two peak risk periods for the onset of eating disorders are early adolescence and late teens (Watson et al, 2010). Funded under ATAPS a young person can access 12 individual sessions and 12 groups per calendar year.

The paper will present the background and rationale for the program, an outline of the evidence based approaches offered and the preliminary data gathered which demonstrates the effectiveness of the interventions.

This abstract was presented in the **Disordered Eating – Characteristics & Treatment** stream of the 2013 ANZAED Conference.

